# Genetic diversity of natural orchardgrass (*Dactylis glomerata* L.) populations in three regions in Europe

**DOI:** 10.1186/1471-2156-14-102

**Published:** 2013-10-29

**Authors:** Luisa Last, Franco Widmer, Wendy Fjellstad, Siyka Stoyanova, Roland Kölliker

**Affiliations:** 1 Agroscope Reckenholz-Tänikon Research Station ART, Reckenholzstrasse 191, Zurich 8046, Switzerland; 2 Norwegian Forest and Landscape Institute, P.O. Box 115, Ås 1431, Norway; 3 Institute of Plant Genetic Resources “K.Malkov”, Sadovo 4122, District Plovdiv, Bulgaria

**Keywords:** *Dactylis glomerata*, SSR, Distinct European grassland, Genetic diversity, Population structure, Rare alleles

## Abstract

**Background:**

*Dactylis glomerata* (orchardgrass or cocksfoot) is a forage crop of agronomic importance comprising high phenotypic plasticity and variability. Although the genus *Dactylis* has been studied quite well within the past century, little is known about the genetic diversity and population patterns of natural populations from geographically distinct grassland regions in Europe. The objectives of this study were to test the ploidy level of 59 natural and semi-natural populations of *D. glomerata*, to investigate genetic diversity, differentiation patterns within and among the three geographic regions, and to evaluate selected populations for their value as genetic resources.

**Results:**

Among 1861 plants from 20 Swiss, 20 Bulgarian and 19 Norwegian populations of *D. glomerata*, exclusively tetraploid individuals were identified based on 29 SSR markers. The average expected heterozygosity (H_E,C_) ranged from 0.44 to 0.59 and was highest in the Norwegian region. The total number of rare alleles was high, accounting for 59.9% of the amplified alleles. 80.82% of the investigated individuals could be assigned to their respective geographic region based on allele frequencies. Average genetic distances were low despite large geographic distances and ranged from D = 0.09 to 0.29 among populations.

**Conclusions:**

All three case study regions revealed high genetic variability of tetraploid *D. glomerata* within selected populations and numerous rare and localized alleles which were geographically unique. The large, permanent grassland patches in Bulgaria provided a high genetic diversity, while fragmented, semi-natural grassland in the Norwegian region provided a high amount of rare, localized alleles, which have to be considered in conservation and breeding strategies. Therefore, the selected grassland populations investigated conserve a large pool of genetic resources and provide valuable sources for forage crop breeding programs.

## Background

*Dactylis glomerata* L. (orchardgrass or cocksfoot), a long-lived and perennial grassland species is the fourth most important forage grass in the world [1]. Its economic value is based on its high productivity and its disease resistance under varying climatic conditions [2]. Due to its high forage quality, i.e. sugar and protein contents, shade tolerance and persistence; the species *D. glomerata* is used for hay or silage production and grazing worldwide. Continuous outcrossing by wind-pollination, natural selection and adaptation processes have resulted in a wide geographic range and large morphological variability [3]. *D. glomerata* has a genome size of 4312 Mbp and comprises diploid (2n = 2× = 14), tetraploid (2n = 4× = 28) and hexaploid (2n = 6× = 42) accessions [1]. Polyploidy in this complex is known to result from auto-polyploidy due to polysomic inheritance [4], which can reduce the loss of genetic variation within populations [5]. Within natural populations and among the more than 200 cultivars currently available, tetraploid *D. glomerata* are the most widespread [1]. However, diploid and tetraploid populations have been recorded living in sympatry, e.g. on the Iberian peninsula [6]. Sympatric appearance can either result from habitat changes leading to the intermixing of diploid and tetraploid populations, the formation of auto-tetraploids or hybridization among individuals of different ploidy levels [7]. Compared to tetraploid populations, hexaploid populations of *D. glomerata* are rare and restricted to certain areas, e.g. Libya, Egypt or Spain [8]. Polyploid populations are of major importance in nature. They have evolutionary benefits due to their increased heterozygosity and decreased inbreeding depression. They are able to more easily colonize new niches and capable of coping with changing ecological conditions on a broad geographical range [9]. Tetraploid individuals are also characterized by a great genetic variability and an increased cell, ligule and plant size [10].

The high plasticity and heterogeneity of the genome of *D. glomerata* has led to a widespread occurrence in natural and semi-natural grassland across Europe. Natural populations of *D. glomerata* are of major importance for forage crop breeding. In natural and semi-natural grasslands, those populations harbor high genetic diversity, which provides advantages for future breeding and conservation programs in particular with respect to climatic changes and an increasing demand for forage and food production [11]. Detailed information on genetic diversity of natural populations of *D. glomerata*, which could be sources for genetically diverse material, is rather scarce. Investigation of genetic patterns in natural or semi-natural grassland populations may not only reveal fundamental knowledge on population genetic structures, but may also support the evaluation and utilization of natural resources with respect to forage crop improvement and *in situ* conservation. Our recent investigations in Switzerland suggest high genetic diversity within, but low genetic variability among populations in permanent grassland (Last et al., submitted).

Geographically distinct populations can differ in their level of genetic diversity or in the distribution of diversity within and among regions [12]. The value of separated geographic regions for forage crop improvement arises from the limited gene flow among those populations and their independent developement under different conditions [13]. These distinct sites are differentiated by various environmental factors such as soil conditions, average temperature or day length and may contain populations harboring valuable traits or alleles that could be used in future breeding programs [14]. This diversity and variability from different geographic regions could be used for *in situ* protection of forage crop genotypes and populations from genetic erosion and provide new germplasm for forage crop breeding.

Simple Sequence Repeats (SSRs) are genetic markers consisting of one to six nucleotides occurring in a repeated pattern (tandem repeats). Their high abundance across the genome, neutral and co-dominant inheritance, and highly polymorphic character qualify SSRs as multi-allelic genetic markers for a broad range of applications, e.g., in breeding and crop improvement as well as in population and ecological genetics (summarized by Kalia et al. [15]).

The aim of this study was to investigate the population structure and genetic variability of natural and semi-natural *D. glomerata* populations in Bulgaria, Norway and Switzerland, representing three grassland regions in Europe. The objectives were: (1) to investigate the ploidy level of *D. glomerata* individuals in three selected grassland regions, (2) to study the genetic diversity and patterns of differentiation within and among populations from different geographical regions, (3) to evaluate the use of geographically distinct regions for the *in situ* conservation of genetic resources of the grass species *D. glomerata*.

## Methods

### Sampling sites and plant material

Sampling sites were located in grassland regions of three European countries. The Bulgarian region (BG) was located in the Smoljan region in the Rhodope Mountains of South Central Bulgaria. At altitudes ranging from 900 to 1400 m.a.s.l, the 20 selected sampling sites were distributed across an area of 3193 km^2^, with distances of 0.18 to 47.36 km between sites (Ø = 1.34 km between sampling sites on farm, Ø = 23.59 km between sites on different farms). Management was characterized by low-input farming of permanent grassland for cattle and sheep based dairy production. The Swiss region (CH) was located in the canton Obwalden, in the Northern Swiss Alps. Ranging from 600 to 1100 m.a.s.l, the 20 selected sampling sites were distributed across an area of 12 km^2^, with distances of 0.09 to 6.03 km between sites (Ø = 0.44 km between sampling sites on farm, Ø = 1.97 km between sites on different farms). Farms were dominated by natural, permanent grassland for cattle-based dairy production (Last et al., submitted). The Norwegian region (NO), located in Nord-Østerdal in the north of Hedmark County, covered an area of 4871 km^2^, ranging from 500 to 1600 m.a.s.l.. The 19 sampling sites, which had not been re-sown for at least 6 years, were located at distances of 0.06 to 46.69 km from one another (Ø = 4.19 km between sampling sites on farm, Ø = 19.35 km between sites on different farms). Management was characterized by sheep raising and hay production. Seed mixtures applied in the Norwegian sampling sites did not contain *D. glomerata* cultivars and occurring *D. glomerata* were considered natural populations. On-farm interviews and questionnaires were used to obtain information about farming systems, on-farm production, management and the potential application of commercial seed mixtures.

Fresh leaf tissue of plant tillers was sampled from randomly selected *D. glomerata* plants from a total of 59 sampling sites (Table 1) during spring and summer 2010. With few exceptions, each population at one sampling site was represented by 32 individuals separated by a distance of at least one meter (Table 1). The collected plant material from each individual was immediately placed in a 15 ml plastic tube half-filled with silica gel, where it was left to dry until DNA extraction.

**Table 1:** **Population characteristics of 59 *Dactylis glomerata *** **populations from three regions in Europe**

**Population**	**n**	**Longitude (°, east)**	**Latitude (°, north)**	**H**_**E,C**_	**A**	**Rare alleles (<5%)**	**Rare alleles per locus (<5%)**
CH01	32	8° 11′ 27.3″	46° 52′ 38.6″	0.53	5.17	48	1.66
CH02	32	8° 11′ 30.2″	46° 52′ 40.7″	0.56	5.65	62	2.14
CH03	32	8° 11′ 35.4″	46° 53′ 07.3″	0.53	5.51	64	2.21
CH04	32	8° 11′ 32.7″	46° 53′ 10.0″	0.53	5.06	45	1.55
CH05	32	8° 11′ 27.1″	46° 52′ 51.3″	0.52	5.27	56	1.93
CH06	32	8° 10′ 52.3″	46° 52′ 20.4″	0.54	5.55	62	2.14
CH07	32	8° 11′ 34.0″	46° 52′ 54.9″	0.52	4.89	44	1.52
CH08	32	8° 10′ 49.2″	46° 53′ 00.0″	0.51	5.06	47	1.62
CH09	32	8° 12′ 27.4″	46° 53′ 14.6″	0.53	5.03	45	1.55
CH10	32	8° 12′ 34.9″	46° 53′ 44.5″	0.55	5.48	60	2.07
CH11	32	8° 09′ 55.2″	46° 52′ 23.7″	0.54	5.10	48	1.66
CH12	32	8° 10′ 02.4″	46° 52′ 21.2″	0.53	5.34	61	2.10
CH13	32	8° 11′ 48.9″	46° 52′ 56.5″	0.55	5.34	53	1.83
CH14	32	8° 11′ 51.5″	46° 52′ 50.0″	0.55	5.72	62	2.14
CH15	32	8° 14′ 11.7″	46° 53′ 48.5″	0.53	5.20	53	1.83
CH16	32	8° 13′ 15.7″	46° 53′ 24.1″	0.52	5.34	54	1.86
CH17	32	8° 10′ 13.0″	46° 52′ 07.0″	0.53	5.00	39	1.34
CH18	32	8° 10′ 16.8″	46° 52′ 03.9″	0.53	5.17	53	1.83
CH19	32	8° 11′ 21.9″	46° 53′ 18.0″	0.53	5.55	57	1.97
CH20	32	8° 11′ 08.2″	46° 52′ 53.7″	0.53	5.51	61	2.10
BG01	32	24° 40′ 15.1″	41° 35′ 38.2″	0.52	5.41	51	1.76
BG02	32	24° 40′ 17.2″	41° 35′ 31.4″	0.54	5.17	42	1.45
BG03	32	24° 16′ 04.8″	41° 40′ 48.3″	0.47	4.69	47	1.62
BG04	32	24° 16′ 46.3″	41° 40′ 51.4″	0.44	4.44	37	1.28
BG05	32	24° 28′ 49.0″	41° 53′ 35.8″	0.55	5.44	56	1.93
BG06	32	24° 28′ 41.3″	41° 53′ 57.7″	0.53	5.03	42	1.45
BG07	32	24° 31′ 44.1″	41° 40′ 33.1″	0.53	5.48	59	2.03
BG08	32	24° 31′ 16.6″	41° 39′ 37.7″	0.53	5.37	49	1.69
BG09	32	24° 35′ 47.8″	41° 39′ 03.8″	0.50	5.24	45	1.55
BG10	32	24° 34′ 08.0″	41° 38′ 54.7″	0.56	5.69	59	2.03
BG11	32	24° 47′ 16.2″	41° 40′ 15.9″	0.51	4.75	38	1.31
BG12	32	24° 47′ 00.3″	41° 42′ 25.3″	0.57	5.62	53	1.83
BG13	33	24° 46′ 52.5″	41° 32′ 46.1″	0.51	4.93	42	1.45
BG14	32	24° 45′ 41.0″	41° 31′ 46.3″	0.48	4.79	40	1.38
BG15	33	24° 44′ 04.1″	41° 33′ 42.6″	0.59	6.13	69	2.38
BG16	32	24° 44′ 09.1″	41° 33′ 38.0″	0.55	5.34	53	1.83
BG17	31	24° 44′ 37.3″	41° 37′ 06.9″	0.54	5.13	50	1.72
BG18	32	24° 44′ 27.8″	41° 37′ 08.2″	0.47	4.37	40	1.38
BG19	32	24° 43′ 46.9″	41° 49′ 50.7″	0.49	4.72	32	1.10
BG20	32	24° 44′ 04.3″	41° 49′ 42.5″	0.53	5.24	45	1.55
NO01	32	10° 59′ 13.0″	62° 24′ 14.0″	0.54	4.96	42	1.45
NO02	32	11° 02′ 06.4″	62° 26′ 16.9″	0.55	4.82	40	1.38
NO03	31	10° 51′ 46.8″	62° 25′ 03.1″	0.55	4.65	41	1.41
NO04	32	10° 47′ 57.6″	62° 25′ 21.7″	0.57	5.06	46	1.59
NO05	32	10° 51′ 15.0″	62° 25′ 01.7″	0.54	4.89	47	1.62
NO06	32	10° 45′ 01.2″	62° 24′ 47.7″	0.55	4.58	38	1.31
NO07	32	10° 50′ 27.4″	62° 25′ 22.5″	0.55	4.93	42	1.45
NO08	32	10° 47′ 01.8″	62° 28′ 17.7″	0.55	5.41	57	1.97
NO09	32	10° 50′ 17.6″	62° 25′ 23.1″	0.57	5.24	47	1.62
NO10	32	10° 46′ 13.5″	62° 26′ 09.3″	0.54	4.44	33	1.14
NO11	32	11° 12′ 41.1″	62° 29′ 35.6″	0.54	4.48	33	1.14
NO12	32	11° 06′ 58.1″	62° 28′ 27.1″	0.54	4.96	43	1.48
NO13	32	11° 11′ 29.8″	62° 30′ 09.5″	0.54	4.79	40	1.38
NO14	6	11° 11′ 04.6″	62° 29′ 49.2″	0.51	3.55	6	0.21
NO15	32	11° 19′ 41.7″	62° 28′ 45.9″	0.56	5.13	53	1.83
NO16	31	10° 43′ 00.1″	62° 16′ 35.1″	0.56	5.65	63	2.17
NO17	32	10° 38′ 51.8″	62° 20′ 40.0″	0.55	5.10	50	1.72
NO18	32	10° 48′ 07.5″	62° 08′ 21.6″	0.53	4.65	40	1.38
NO19	32	10° 48′ 11.5″	62° 08′ 21.8″	0.53	4.86	41	1.41

The number of individuals (n), geographical coordinates, expected heterozygosity (H_E,C_), allelic richness (A), the total number of rare alleles and the mean number of rare alleles per locus are given per population. Twenty populations originated from Switzerland (CH), 20 from Bulgaria (BG) and 19 from Norway (NO).

### DNA extraction and SSR analysis

Dried plant tissue (30 mg) was ground three times by metal beads at 30 hz using a mill (Retch GmbH, Haan, Germany). DNA was extracted using the NucleoSpin® 96 Plant II (Marchery-Nagel, Düren, Germany) extraction kit. Quantity and quality of DNA was assessed by photospectrometry using NanoDrop (Thermo Fisher Scientific, Wilmington, DE, USA) and the ND-1000 software. SSR marker analysis was performed in multiplex reactions using 29 primer pairs (Table 2) [16,17] on all 1861 *D. glomerata* individuals. The PCR assays were conducted in a volume of 20 μl containing 15 ng of genomic DNA, 0.25 U DNA polymerase, 1× GoTag® Flexi Buffer, 3 mM MgCl_2_, 200 μM dNTPs (Promega, Madison, WI, USA) and 0.2 μM of fluorescently labeled forward primers (FAM, HEX, ATTO550, synthesized by Microsynth, Balgach, Switzerland) and unlabeled reverse primers. PCR was performed using an iCycler (Bio-Rad Laboratories, UK) under the following conditions: Initial denaturation at 94°C for 5 min, followed by 12 cycles of 'touchdown’ PCR consisting of 30 s denaturation at 94°C and 1 min annealing between 72°C and 60°C (decreased 1°C at each cycle), 1 min at 60°C, 1 min elongation at 72°C, followed by 25 cycles denaturation for 30 s at 94°C, 1 min at 60°C, 1 min elongation at 72°C and a final extension of 15 min at 72°C. Fragments were sized on a 48 capillary 3730×l DNA Analyzer using POP 7 polymer and the ROX HD400 standard (Applied Biosystems, Foster City, CA, USA). Fragment analysis was performed at the Genetic Diversity Centre (GDC, ETH, Zurich, Switzerland). SSR alleles were automatically binned using GeneMarker® Version 1.95 (SoftGenetics LLC®, State College, PA, USA). All binned peaks were checked for correct assignment to corresponding bands and corrected manually. Samples were randomly arranged for PCR and fragment analysis.

**Table 2:** Marker characteristics for the 29 simple sequence repeat (SSR) markers used in this study

				**Total number of**				
**Marker name**	**Size range**	**Repeat**		**Alleles**	**Rare alleles**	**Alleles per region**		
	**rmu**	**motif**	**PIC**		**< 5%**	**CH**	**NO**	**BG**
^1^A01C20	115-141	2	0.61	13	11	13	12	13
^1^A01I13	146-168	2	0.78	12	9	10	9	12
^2^Dg_Contig66	161-203	3	0.76	14	10	12	10	11
^2^BG04056B2F02_r1	83-140	3	0.79	20	17	17	17	19
^2^Dg_Contig330	166-184	3	0.71	7	4	7	7	6
^2^BG04059A1A07_f1	73-136	3	0.88	23	19	21	21	20
^2^Dg_Contig3046	208-226	3	0.73	7	4	7	7	7
^2^Dg_Contig5978	219-234	3	0.69	6	3	6	6	6
^2^Dg_Contig4556	189-225	3	0.77	13	9	12	13	12
^2^Dg_Contig10135	209-224	3	0.72	6	3	6	6	6
^2^Dg_Contig660	95-119	3	0.58	9	6	9	8	9
^2^BG04030A2C10.f1	156-192	3	0.72	12	9	12	12	12
^2^Dg_Contig10764	81-99	3	0.54	7	5	5	6	7
^2^Dg_Contig4296	184-214	3	0.78	11	7	11	11	9
^2^Dg_Contig4110	179-197	3	0.61	7	4	6	7	7
^2^Dg_Contig6373	174-195	3	0.67	8	5	7	6	8
^2^BG04046A2B07.f1	209-242	3	0.83	12	9	12	12	12
^2^Dg_Contig12453	90-110	2	0.81	11	6	11	10	10
^2^Dg_Contig10487	160-178	3	0.76	7	4	7	7	7
^2^Dg_Contig4921	254-260	3	0.50	3	1	2	3	3
^2^Dg_Contig4563	115-130	3	0.61	6	4	5	6	6
^2^Dg_Contig10236	94-104	2	0.52	6	3	5	6	6
^2^BG04035A2D08.r1	117-129	3	0.50	7	5	7	7	7
^2^Dg_Contig3264	135-138	3	0.45	2	0	2	2	2
^2^Dg_Contig11508	179-188	3	0.25	4	3	4	3	3
^2^Dg_Contig12217	93-129	3	0.37	11	9	9	8	10
^2^Dg_Contig4478	216-231	3	0.50	5	3	5	4	4
^2^Dg_Contig667	184-196	3	0.12	5	4	5	5	4
^3^Dg_Contig1483	99-107	4	0.30	3	1	3	3	3

Marker names, size range in relative migration units (rmu’s), repeat motif, PIC (Polymorphic Information Content) and allele data of 29 simple sequence repeat (SSR) markers are given based on 59 populations of *Dactylis glomerata* from three European regions. Primer sequences were published by Xie et al. [17]^1^, Bushman et al. [16]^2^and personally communicated by Shaun Bushman (F: TGGACTACATGATGAACCAGTACC, R: GGTTCTCTTCCATGCTCATGTT)^3^.

### Statistical analysis

The ploidy level of collected individuals was calculated based on the maximum and mean number of alleles per locus, for all samples and across all loci using the R package “polysat” [18,19]. As used in the study of Aerts et al. [20] and also proposed by Palop-Esteban et al. [21], the R package “polysat” provided a useful statistical tool to handle microsatellite data while considering tetraploidy within populations. The total number of alleles per locus and the polymorphic information content (PIC) were calculated for each primer (Table 2). Genetic diversity of *D. glomerata* within populations was estimated using the unbiased measurement of average expected heterozygosity corrected for sample size H_E,C_[22] and allelic richness (A, mean number of alleles per locus) per population (Table 1). H_E,C_ was calculated based on the tetraploid data set using the ATETRA program 1.3a [23]. The total number of rare alleles, defined as alleles with a frequency < 0.05 per locus were calculated, and rare alleles were classified in allele categories (Table 3) as proposed by Brown [24]. A multiple comparison of diversity indices among regions was conducted using the Tukey's HSD (honestly significant difference) test. Genetic structure and variation among regions, among populations and among individuals was assessed by Analysis of Molecular Variance (AMOVA) using the R package “vegan” [25,26]. AMOVA was based on pairwise Euclidean distance using binary SSR allele data. Genetic differentiation between populations was addressed by the calculation of pairwise genetic distance D [27] based on allele frequencies using the R packages “polysat” and “adegenet” [18,28]. Mantel test was performed to test for correlation among matrices of pairwise genetic distance (D) and the respective geographic distance (km) in order to test for isolation-by-distance (IBD) applying the Isolation by Distance Web Service Version 3.23 (IBDWS, http://ibdws.sdsu.edu/, [29]). Significance of correlation was tested by 999 random permutations. Population genetic variation was investigated by principal component analysis (PCA) based on a binary data matrix (present/absent) derived from SSR alleles. Spatial population structure and membership of individuals to populations were investigated based on the binary data set using the model-based clustering method implemented in the STRUCTURE program version 2.3.1 [30]. The optimum number of subpopulations (*K*) among and within regions was calculated based on six independently repeated runs of 100000 iterations (length of burn-in period) followed by 100000 Markov Chain Monte Carlo (MCMC) repetitions after burn-in applying the implemented admixture model and correlated frequencies [31]. For the estimation of subpopulations among regions, *K* was set from 1 to 8. Within regions, *K* was set from 1 to 20, (19 in the Norwegian region). The *K*-value revealing the highest maximum likelihood 'Ln P(D)’ after several independent runs was selected for the assignment of individuals to subpopulations based on their membership probability. Populations in which all individuals had membership probability of ≥ 0.8 were regarded as distinct populations, whereas populations containing individuals with membership probability < 0.8 were considered as admixed [32]. In order to keep the high number of polymorphic loci and in order to consider also rare alleles for the data analysis, we decided to apply the commonly used and more conservative approach based on binary allele scoring for AMOVA, isolation by distance, PCA analysis and the analysis by STRUCTURE. This approach is generally accepted to investigate synthetic and natural populations [20,33-35].

**Table 3:** Classification of the total and average number of alleles in the three European regions

**Allele categories**	**CH**	**NO**	**BG**
Common (>5%), widespread (>2 locations)	120	119	136
Common (>5%), sporadic (2 locations)	8	8	9
Common (>5%), localized (1 location)	18	21	26
Rare (<5%), widespread (>1 location)	75	61	55
Rare (<5%), localized (1 location)	18	25	15
Total number of alleles	239	234	241
Unique alleles per region	5	2	7
Mean number of alleles per locus	8.20	8.10	8.31
Mean number of alleles per locus per population	5.30	4.85	5.15
Mean number of rare alleles per locus per population	1.85	2.21	1.65

Correspondences to categories were based on the percentage of occurrence of alleles above (common allele) and below (rare allele) the five per cent mark, as well as the number of populations (locations) in which the allele was detected within the Swiss (CH), Norwegian (NO) and Bulgarian (BG) region.

## Results

### Ploidy level and genetic diversity within populations

**Figure 1:**
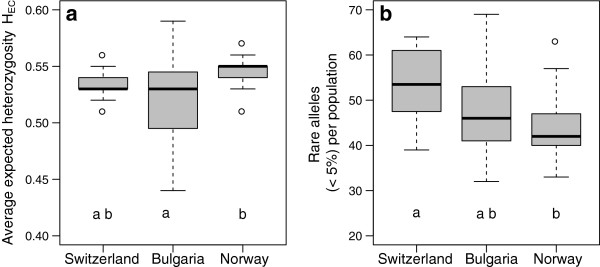
**Average expected heterozygosity (H**_**E,C**_**) and the total number of rare alleles in populations of *Dactylis glomerata*.** Boxplots of SSR data of 1861 individuals from 59 *Dactylis glomerata* populations, showing the median, 25% and 75% quartile (box) of **(a)** average expected heterozygosity values (H_E,C_) and **(b)** the total number of rare alleles (< 5% occurrence) per population for three European regions (Switzerland, Bulgaria and Norway). Boxes indicated with different letters are significantly different (*P *< 0.05) on the basis of pairwise comparison using Tukey’s honestly significant difference test.

The maximum and mean number of alleles per locus across all loci was ≥ 3 for each sample. Consequently, at least one out of 29 loci per individual revealed 3 to 4 alleles per locus, which indicated tetraploidy of the corresponding individual (data not shown). Among 1861 *D. glomerata* plants, the 29 SSR primers detected 257 polymorphic alleles, varying in size from 73 to 260 bp (Table 2). The polymorphic information content (PIC) varied considerably, ranging from 0.12 to 0.88 (mean: 0.62 ± 0.18) (Table 2). The average expected heterozygosity (H_E,C_) across all loci was high in all regions, ranging from 0.44 to 0.59 (Table 1). The greatest variation in H_E,C_ was detected in Bulgaria (Figure 1a). The mean H_E,C_ was significantly higher in the Norwegian region (H_E,C_ = 0.54) when compared to the Bulgarian region (H_E,C_ = 0.52, *P* < 0.05). There was no significant difference between Switzerland (H_E,C_ = 0.53) and the two other regions (Figure 1a). The total number of rare alleles (frequency < 5%) across all loci was 154 and covered 59.9% of all amplified allelic bands across all the three regions. 103 of the amplified alleles were classified as common with an occurrence larger than 5% and in more than two locations. Not all alleles were detectable in all of the regions. Two to seven unique alleles within one region were detected (Table 3).

The Bulgarian region had the greatest total number of alleles (241) (Table 3). The mean number of rare alleles per population within the Swiss region was significantly higher than within the Norwegian region (Tukey HDS, *P* < 0.05) (Figure 1b). Within the Swiss region, the highest mean number of alleles and rare alleles per locus per population (5.30, 1.85) was detected, which was significantly different from Norway (Tukey HDS, *P* < 0.05), but not Bulgaria (Table 3). Hierarchical analysis of molecular variance (AMOVA) across individuals from all three regions revealed most of the genetic diversity to be due to variation within populations (86.43%), while the variation among regions (6.46%), among farms within regions (3.88%) and among populations within farms (3.22%), was small but significant (Table 4). This pattern of within and among population partitioning of genetic variation was representative for *D. glomerata* populations in all three regions (Table 4).

**Table 4:** **Analysis of Molecular Variance (AMOVA) of 20 Swiss, 20 Bulgarian and 19 Norwegian *Dactylis glomerata *** **populations**

**Source of variation**	**DF**	**Percentage of variation (%)**
**(Switzerland)**		
Variation among farms	9	1.923
Variation among populations within farms	10	2.019
Variation within population	620	96.059
Total	639	100
**(Bulgaria)**		
Variation among farms	9	7.365
Variation among populations within farms	10	5.78
Variation within population	620	86.855
Total	639	100
**(Norway)**		
Variation among farms	9	2.637
Variation among populations within farms	9	2.114
Variation within population	561	95.249
Total	579	100
**(All three European regions)**		
Variation among regions	2	6.466
Variation among farms within regions	27	3.881
Variation among population within farms	29	3.22
Variation within population	1802	86.434
Total	1860	100

Analysis was performed using a binary data set based on 29 SSR markers which generated 257 SSR bands. All variance components were significant (*P *< 0.001) based on 999 permutations.

### Genetic distances among populations

**Figure 2:**
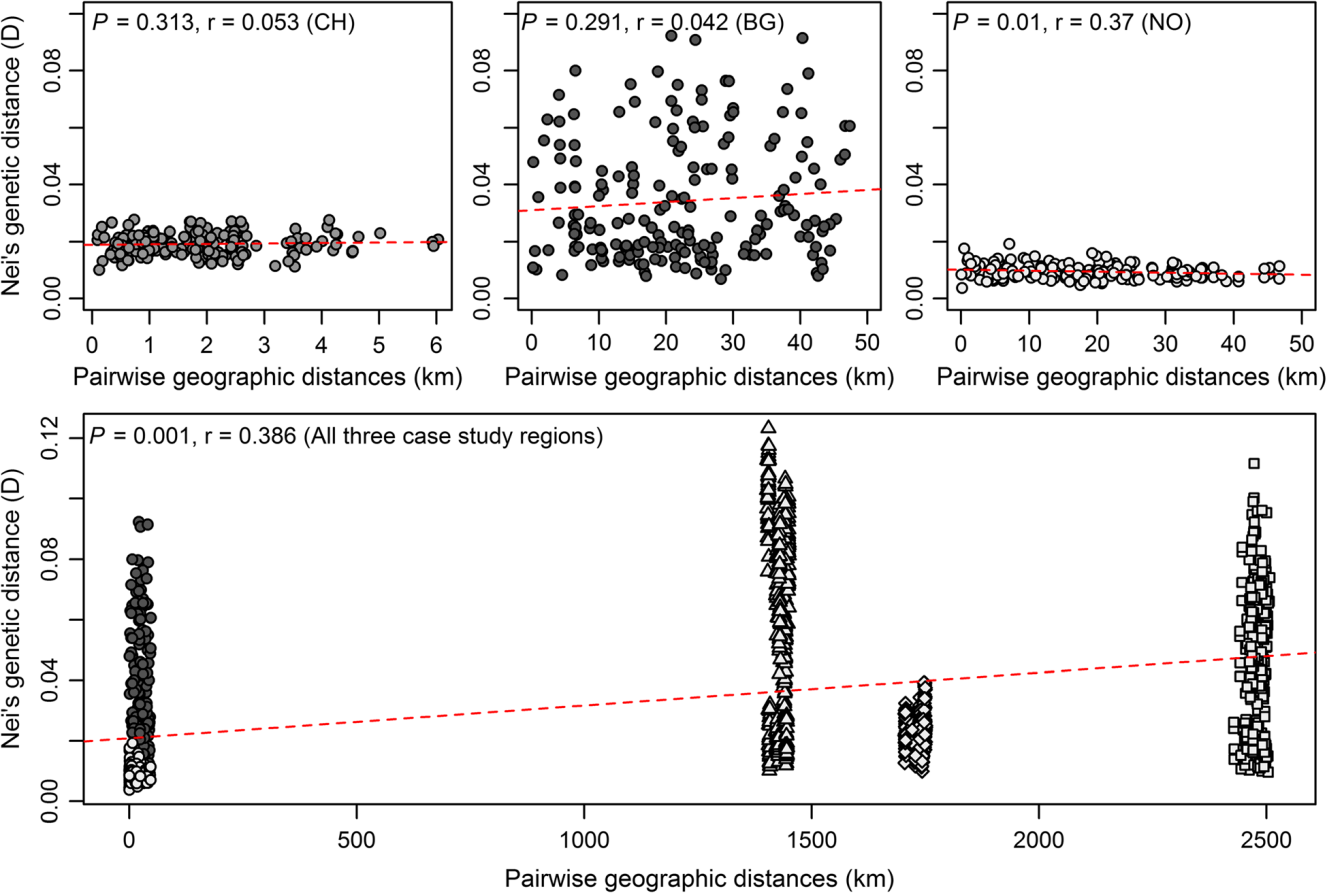
**Isolation by distance (IBD) between 59 *Dactylis glomerata *populations within and among three European regions.** IBD was based on the Mantel test with 999 random permutations for and between 20 Swiss (CH), 20 Bulgarian (BG) and 19 Norwegian (NO) populations of *Dactylis glomerata*. Filled circles represent pairs of geographic and genetic distances within three European regions (dark grey (BG), grey (CH), light grey (NO)), unfilled symbols are pairs among regions (triangles (CH – BG), diamonds (CH – NO), squares (BG – NO)).

Pairwise genetic distances were low to moderate for all pairs of regions, ranging from D = 0.03 (CH-NO) and D = 0.06 (NO-BG), to D = 0.09 (CH-BG). Genetic distances among populations within regions ranged from D = 0.01 to 0.02 (CH), D = 0.009 to 0.05 (NO) and D = 0.01 to 0.21 (BG). The greatest genetic distance between populations from different regions was D = 0.29 for the Swiss population CH04 and the Bulgarian population BG04. Significant correlations between pairwise genetic distances (D) and the corresponding geographical distances between populations within the Norwegian region (r_M_ = 0.37, *P* = 0.01) and among the three regions (r_M_ = 0.39, *P* < 0.001) were identified by testing for isolation by distance (Figure 2).

### Population structure

**Figure 3:**
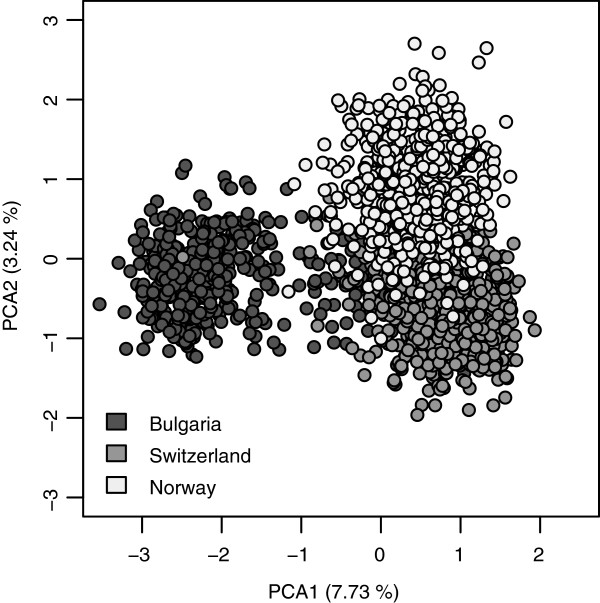
**Principal component analysis on 1861 genotypes from three European regions using 29 SSR markers.** PCA 1 and PCA 2 refer to the first and second principal component, respectively. The corresponding percentages refer to the proportion of variance explained by the axes. Coloration is according to region (dark grey (Bulgaria), grey (Switzerland), light grey (Norway)).

**Figure 4:**
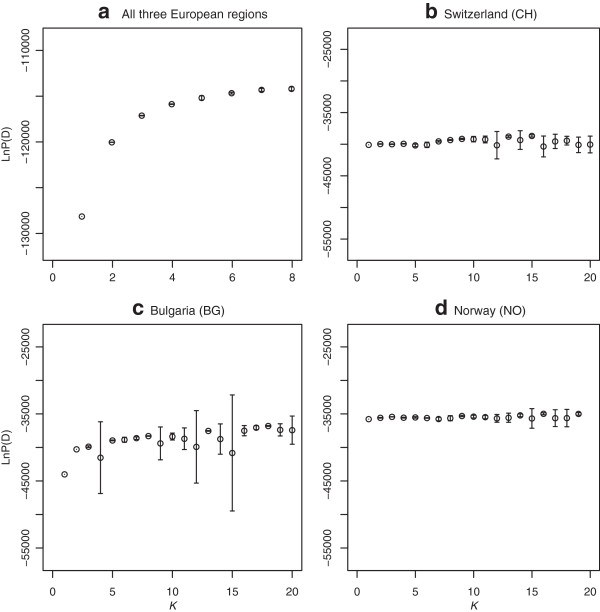
**Log probability of *K *being the number of *Dactylis glomerata *populations detected.** Plot of the mean (± SD) of the natural log probability of the data [LnP(D)] over 6 repetitive STRUCTURE runs of **a)***K* = 1-8 for all three regions; **b/c)***K* = 1-20 for Switzerland and Bulgaria, and **d)***K* = 1-19 for Norway.

**Figure 5:**
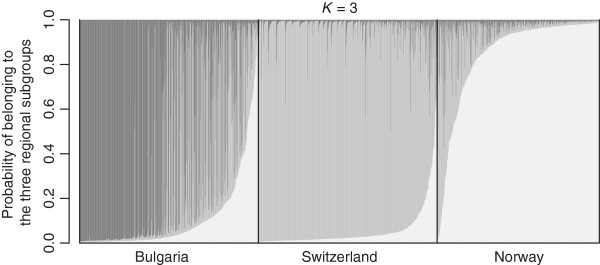
**Inferred ancestry of 1861 *Dactylis glomerata *individuals in 59 populations from three regions using STRUCTURE.** Each individual genotype is represented by a thin bar from 0.0 to 1.0, where the bar is colored according to the probability of belonging to each of the three subgroups (dark grey (Bulgaria), grey (Switzerland), light grey (Norway)).

A moderate but clear separation of genotypes among regions was revealed by principle component analysis (PCA) based on 1861 individuals and 257 SSR alleles (Figure 3). The first two principle components (PCs) explained 10.97% of the total molecular variation among samples, while the third PC explained less than 2%. For *D. glomerata* from different regions, the number of populations *K* = 3 revealed greater variability of maximum likelihood (Ln P(D)) among different tested *K* values than among repeated runs and was considered as the optimal number of populations (Figure 4a). In total, 1504 of 1861 individuals were assigned to one of the three populations due to their membership probability ≥ 0.8. The proportion of membership in each pre-defined cluster (Bulgaria, Switzerland and Norway) was greatest in Switzerland (91.2%), followed by Norway (84.5%). Only 62.2% of the individuals from Bulgaria were assigned exclusively to the corresponding cluster (Figure 5). For population structures within regions, no definite number of populations could be defined based on selected numbers of *K* (Figure 4b - d).

## Discussion

### Ploidy level of *D. glomerata* populations

This study revealed exclusively tetraploid individuals of *D. glomerata* sampled from 59 natural and semi-natural populations in three distinct regions of Europe. Although tetraploid and diploid populations can occur in sympatry [6,7], autotetraploid individuals of *D. glomerata* have been reported to be most abundant in cultivars and natural populations [36], which was clearly supported by this study. This provided first information about the value of selected populations considering polyploidy as an important factor for forage crop breeding. These tetraploid populations could be potential sources increasing forage quality and yield, which is often related to tetra-ploidy in forage grasses. Furthermore, these exclusively tetraploid populations usually contain higher genetic diversity compared to diploid populations as has been shown for *Rorippa amphibian* or *Bromus* species in European populations [37,38]. Both, tetraploidy and the corresponding high degree of genetic diversity identified within selected populations of *D. glomerata* indicated them to be valuable sources for germplasm collections.

### Genetic and allelic diversity within populations

The genetic diversity in terms of average expected heterozygosity H_E,C_ of *D. glomerata* within populations from different grassland regions in Europe was comparable with *D. glomerata* cultivars [39]. The genetic diversity of populations is a major capacity for the adaptation to various and changing environmental conditions [40]. However, this study showed that most of the genetic variation was detected within, rather than between populations, as it has been demonstrated for other agriculturally important grass species such as *Poa alpina*, *Festuca pratensis* or *Lolium multiflorum*[32,41]. Similarly, the variation detected within geographic regions was larger than the variation between them, which is congruent with studies on the germplasm of *Lolium perenne* from different geographical regions worldwide [12]. High genetic diversity within *D. glomerata* populations strongly depends on various life history traits, such as the outbreeding mating system and efficient pollen dispersal by wind [42]. On average, the highest H_E,C_ and the lowest mean number of alleles per locus per population within selected populations was detected in the Norwegian region. Since the semi-natural grassland populations did not receive any seed mixtures that included *D. glomerata* varieties, genotypes from natural and commercial gene pools must have immigrated from outside populations [43]. In the Norwegian region, H_E,C_ was high for all populations. As revealed by previous studies on grassland genetic diversity in space and time, habitat age, connectivity and past use in a landscape and historical context have a major impact on current genetic diversity patterns [44]. In the 1950s, the area in Nord-Østerdal was much more open resulting in an high gene flow among connected grassland - hence the low range of H_E,C_[45]. When farming declined in the area, establishing forests disconnected populations and interrupted gene flow among populations [46]. Relatively recent mutations within *D. glomerata* populations could then explain these fragmented grassland patches and the relatively high number of rare and localized alleles. Whereas in the Bulgarian region, some populations of *D. glomerata* revealed high H_E,C_ and others indicated low H_E,C_-values, lowering the average H_E,C_ across all population, but augmenting the range of H_E_ within the region. There, the landscape provided some large grassland patches with high levels of gene flow and high H_E,C_ as well as single, remote grassland patches with low gene flow due to low connectivity. A lower gene flow into more isolated populations has been revealed for grassland species such as *Globularia bisnagarica*[47]. Therefore, in the Norwegian region, collections could quickly capture the genetic variation (except for those rare, localized genes). In Bulgaria more individuals would have to be sampled, but would eventually provide a higher total genetic diversity. Presumably, the Bulgarian populations could be more resilient to environmental changes, because some individuals might have a favorable genotype, whilst in the Norwegian region there is little difference between individuals and, therefore, less possibility for adaptation [48]. The Swiss populations revealed the same small range of H_E,C_ and a lower average H_E,C_ across all investigated populations as the Norwegian region. Here, permanent grassland has been established for a long time without sod disturbances, e.g. rotational forage crops, or the introduction of new genetic material, e.g. by re-sowing or extended seed-recruitment. Furthermore, the selected populations were located in a small geographic range (Last et al., submitted) increasing the connectivity and gene flow among populations leading to constant intermixing and, therefore, high H_E,C_ in all *D. glomerata* populations.

Although, H_E,C_ represents a common measurement for genetic diversity based on allele frequencies, allelic diversity or allelic richness plays a more relevant role for genetic conservation [49]. The presence of many rare alleles and, especially, alleles that were detected in only single populations or regions indicated the potential value of every single population as a genetic resource. A comparable number of rare alleles within grassland species has been detected for *F. pratensis* in Swiss ecotype populations by using SSR markers [32]. In the Norwegian region, grassland sampling sites were fragmented by forests which represents a common landscape structure within this area. Within these fragments, the high selection pressure of fragmentation resulted in increasing genetic differentiation and the loss of rare alleles on the long run [50,51]. The consideration of those natural populations for *in situ* conservation and germplasm collections might comprise the potential to increase the quality of grassland cultivars in terms of resilience and persistence in currently unfavorable areas [52,53].

### Genetic diversity among populations

Although the genetic diversity was high in selected populations and regions, the genetic distance of individuals among populations was low and did not indicate clear distinction of selected populations within regions. These results support previous studies on *D. glomerata* and *L. multiflorum* populations, which investigated populations less than 100 km apart [32,36] (Last et al., submitted). A high degree of gene flow is very common in self-incompatible and wind-pollinating grass species, leading to low genetic distance among individuals and populations [24]. The high abundance of individuals per species may increase gene flow within study sites as revealed for *F. pratensis*[54]. The impact of differentiated evolutionary processes affecting the genetic structure of distinct grassland populations increases with increasing genetic distance due to lacking structural and functional connectivity among populations [46,55]. No isolation by distance was detected in the Swiss region. There, the selected populations originated from a small geographic range with small distances between populations. However, isolation by distance occurred within the Norwegian region where distances among selected populations were high and collection sites scattered on a large geographic area. It may be the fragmentation of the grassland patches and disconnection by landscape change that has led to gene flow restriction, as it has been revealed for *D. glomerata* populations in Turkey [36,44]. In contrast to the Norwegian region, no isolation by distance was detected among the Bulgaria populations. Although this region was of large geographic range, grassland patches (farmland patches) belonging to a single farm were less scattered.

The genetic distance that we found among populations from distinct and distant regions in Europe was slightly higher than the genetic distance between populations within regions, and reflects results from population genetic studies among natural populations and cultivars of *D. glomerata* worldwide [56]. The increase of genetic distance among population of *D. glomerata* between distant regions reflects what has been found in previous studies on isolation-by-distance patterns of *Festuca arundinacea* investigated within large geographic ranges [57]. Although self-incompatible and wind-pollinating species are expected to reach the highest rate of gene flow among individuals and populations, the distance of pollen distribution is restricted and does very rarely reach long-distance transport [54,58]. Although there was a clear separation of populations from distinct geographical regions, the probability of an individual belonging to one of the regional subgroups was less than 80% for some genotypes. This indicated an admixture of genetic information among regions regardless the large geographic distances among regions. These admixtures could either be explained by the assumption of a common ancestor within the Poaceae family and differentiated selective forces resulting from different environmental and ecological conditions [52]. According to this theory, most of the individuals contain the same genotypic constitution adapted to local or geographical conditions, while only single individuals remain admixed as the common ancestors were. Admixture or admixed genotypes might also result from human-mediated transfer of grasses and their seed material among populations that are geographically apart and genetically distinct. A high agricultural importance, the widespread use of common seed material in the past and the constant outcrossing of natural populations and introduced germplasm can additionally affect genetic diversity patterns today [59].

## Conclusions

The investigation of 59 natural and semi-natural populations of *D. glomerata*, not only revealed exclusively tetraploid individuals, but high genetic diversity, a high number of rare and geographically unique alleles in geographically distinct populations. The three regions revealed genetically distinct patterns and were differentiated from each other. These populations of *D. glomerata* might contain valuable sources for plants adapted to specific, but differentiated environmental conditions. Especially the high amount of rare, localized alleles in Norway or the high amount of unique alleles located in Bulgaria may indicate valuable sources for breeding material adapted to climatic and environmental changes in certain regions. To conserve a high amount of genetic diversity large, permanent grassland patches with natural populations of *D. glomerata*, as represented in Bulgaria, should be considered. Fragmented and smaller grassland patches as represented in the Norwegian region on the other hand, can provide a high amount of rare, localized alleles of *D. glomerata.* In general, genetic material from distinct geographical regions and multiple populations should be considered for *ex situ* and *in situ* conservation.

## Competing interests

The authors declare that they have no competing interests.

## Authors’ contributions

LL collected the plant material for this study in Switzerland and Norway, carried out the molecular analysis using SSRs, performed the genetic data analysis and drafted the manuscript. FW discussed the results and participated in writing the manuscript. WF contributed to the sampling site selection and sample collection in Norway. SS participated in the initial discussion of this project, contributed to the sampling site selection and performed the plant material sampling in Bulgaria. WF and SS participated in writing the manuscript. RK conceived and supervised the project, assisted in the data analysis, discussed the results and contributed to draft the manuscript. All authors read and approved the final manuscript.
